# Femoral diaphysis malunion: An overview

**DOI:** 10.1016/j.jcot.2026.103429

**Published:** 2026-04-08

**Authors:** Marco Pes, Yahya Ibrahim, Nikhil Pandit, Alexandros Vris

**Affiliations:** a Royal London Hospital, Whitechapel Road, London, E1 1FR, UK; b Blizzard Institute, Barts and The London School of Medicine and Dentistry, 4 Newark St, London E1 2AT, UK

**Keywords:** Osteotomy [E04.555.580], Bone lengthening [E04.555.120], Orthopedic procedures [E02.718], External fixators [E07.858.442.660]

## Abstract

Femoral diaphyseal fractures are commonly treated with intramedullary nailing; however, malunion remains a clinically significant complication, particularly following delayed presentation, non-operative management, or treatment in resource-limited settings. Femoral diaphyseal malunion may be angular, rotational, longitudinal, or multiplanar and can result in gait disturbance, limb-length discrepancy, patellofemoral overload, secondary joint degeneration, and chronic pain. Given the substantial functional impact, corrective osteotomy is often indicated. This narrative review presents a structured approach to the assessment and management of femoral diaphyseal malunion. Key elements include comprehensive patient evaluation, detailed clinical and radiological deformity analysis, and contemporary operative strategies aimed at restoring alignment, rotation, and limb length while minimising morbidity. Successful correction requires meticulous preoperative planning and careful intraoperative execution, according to available resources. Treatment options include intramedullary nailing, plate fixation, hybrid constructs, and external fixation techniques, each with specific indications. Although circular external fixators allow accurate multiplanar correction, their prolonged use in the femur is poorly tolerated. Fixator-assisted correction has evolved to improve intraoperative control while avoiding long-term external fixation. Modern techniques such as Computer Hexapod-Assisted Orthopaedic Surgery (CHAOS) utilise a temporary intraoperative hexapod frame to achieve precise multiplanar correction, followed by definitive internal fixation within the same procedure. Adjuncts including poller screws, three-dimensional planning, and patient-specific guides further enhance accuracy and stability. Femoral diaphyseal malunion presents complex diagnostic and technical challenges. A systematic, patient-specific approach incorporating modern fixator-assisted and computer-guided techniques enables reliable restoration of alignment and rotation, improving function and quality of life.

## 1 Background

Femoral diaphyseal fractures have an incidence between 10 and 20 per 100,000 people per year.^1^^,^^2^ The mechanism of injury in younger patients is often high energy trauma and may be associated with polytrauma. The preferred treatment involves a closed reduction and intramedullary nailing, allowing early mobilisation. Femoral non-union is less common than in the tibia, due to the good soft tissue coverage and blood supply. Malunion, however, is more common with an incidence of 20-30% but the studies have a low power.^3^ Malunion of the femoral diaphysis is more prevalent in neglected or conservatively managed femoral fractures, particularly in austere or low resource settings.

Resultant deformities can be rotational, angular, longitudinal or multiplanar in nature. If left untreated, femoral malunion may result in symptomatic gait disturbance, patellofemoral overload, secondary knee osteoarthritis, chronic pain or lower back pain.^4^

Given the significant functional impact, corrective osteotomy is often indicated. Successful correction requires a structured evaluation, precise deformity analysis, and careful attention to intraoperative technique. Treatment should be patient and resource specific. This review aims to improve the readers’ understanding of the deformity, clinical assessment and provide advice regarding treatment strategies.

### 1.1 Understanding the patient

**Fig. 1:**
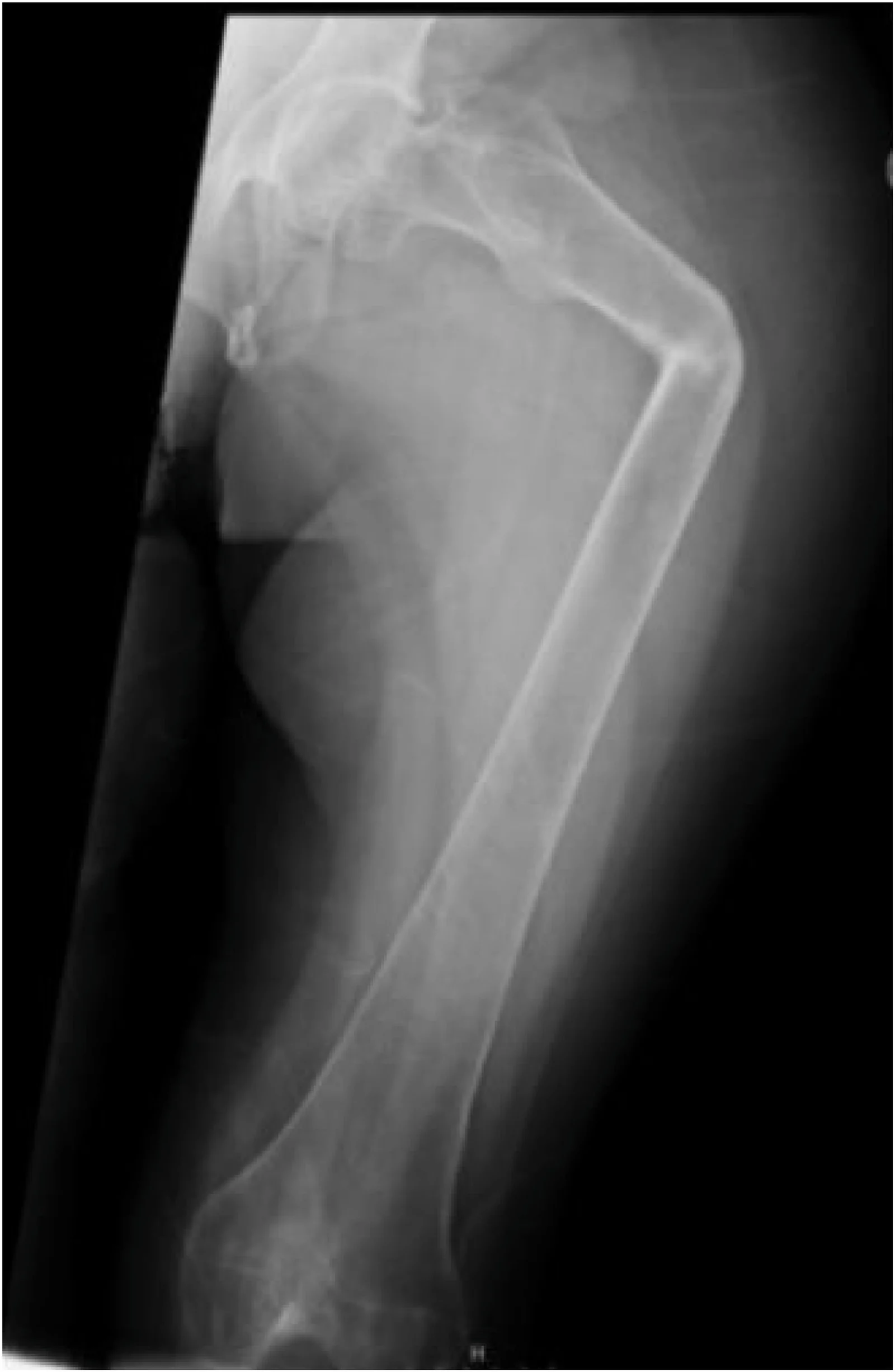
Femoral malunion, with 90° coronal plane deformity in a conservatively treated femur fracture in a patient with Osteomalacia.

Before addressing the deformity surgically, it is important to understand the background of the malunion. Patients who have developed a malunion with or without surgery can be socially and medically complex (Fig. 1). Patients’ socioeconomic circumstances, co-morbidities and safeguarding issues may all be contributing factors. The injury may be self-inflicted, which has been shown to more than double postoperative morbidity.^5^ Non-compliance with postoperative rehabilitation can also cause subsequent treatment failure. Prolonged periods of immobilisation can risk creating other challenges, including bed sores, thromboembolic events, respiratory complications, muscle atrophy and joint contractures.^6^^,^^7^ Their pre-injury level of mobility is also a key factor to consider. It cannot be over-emphasised that a thorough history is key in establishing these facts early in the patient journey. Poor compliance with post operative instructions, age, poor bone quality, co-morbidities, including obesity and congenital or developmental disorders are important factors to consider specifically.

Surgical factors can be categorised as a malreduction, a failure of fixation, or intentional deformity. It is important to interrogate the mitigating circumstances and aetiology. Anatomical factors may make a malreduction more likely,^8^ as well as soft tissue injuries, adjacent fractures, pre-existing metalwork, or deforming forces. The initial fracture pattern may also have played a part in malreduction. Transverse fractures, comminuted fractures and those with bone loss, cannot be reliably reduced without the use of anatomical landmarks proximally and distally (usually femoral anteversion and the distal femoral intercondylar axis). Shortening may also be intentional, in cases of critical bone or soft tissue loss. Gaining access to any imaging from the time of their initial presentation, their intraoperative imaging and any postoperative radiographs is therefore extremely helpful. There may also be human factors responsible for a malunion. Fractures addressed in relatively austere environments, can have suboptimal access to intraoperative imaging, implants and reduction tools, which can also explain how a malunion has occurred. Access to the original operation note can help to explain and better understand any challenges that were faced by the index surgeon. Such factors may be modifiable or non-modifiable when considering treatment options.

### 1.2 Understanding the deformity

Uniplanar deformities may be angular, rotational or longitudinal, but often malunions are multiplanar. Each malunion should be assessed on an individual basis. Clinical assessment is key and should be supplemented with a radiological assessment. If the contralateral limb is normal, then this can be useful to compare against. If this however is not possible, then comparisons should be made with defined population normal ranges.^6^^,^^9^^,^^10^

Clinically significant femoral diaphyseal malunion is generally defined by deformity exceeding accepted anatomical parameters. Angular deformity greater than 5–10° in the coronal or sagittal plane, rotational malalignment greater than 10–15° compared with the contralateral limb, or limb-length discrepancy greater than 1–2 cm have been commonly described as thresholds beyond which symptoms and functional impairment may occur. In particular, several studies have d approximately 15° is associated with altered gait mechanics, anterior knee pain, and functional limitations during activities such as running or stair climbing, and is therefore frequently considered an indication for corrective osteotomy when symptomatic.

However, crucially, the magnitude of the deformity is not the only factor to consider, and slight deviations from normal can be acceptable. The patients' symptoms and overall picture should be taken into account, and comparison can also be made to the patient's contralateral (asymptomatic limb).^11^, ^12^, ^13^, ^14^.

Examination should begin with observation of gait. Angular deformities may reveal themselves as a varus or valgus angulation, or apparent leg length inequality. Apex anterior or posterior angulation may also be complicated by a compensatory fixed flexion or hyperextension of the adjacent joints.^15^ In situations where joints are stiff, one must also assess whether joint degeneration, neurological conditions or soft tissue contractures to tendons, ligaments or skin may be contributing factors.

Leg length inequality may present with secondary pelvic obliquity and scoliosis. In adults, generally leg length inequality is thought to be asymptomatic if less than 2 cm.^16^ If a scoliosis is present, one should check to see if this is a fixed or flexible scoliosis. Several methods exist for measuring LLD including the ‘direct’ method using a tape measure from the Anterior superior iliac spine to the medial malleolus which defines ‘true leg length’ or from the umbilicus to the medial malleolus which defines ‘apparent’ leg length however this can amount to errors. The block method is known as the ‘indirect’ method and accounts for disparity in foot height and deformities. Hanada et al. validated this method of iliac crest palpation and the placement of blocks under the shortened limb to match the contralateral limb.^17^ ^18^ Leg length inequality should be assessed both with Galeazzi testing and Bryant's triangle. Angular deformities may create an apparent limb-length discrepancy, and this must be considered during preoperative imaging and deformity planning.

Rotational deformities of the femoral diaphysis will create adverse biomechanics proximally and distally. This can be performed by assessing the rotational range of motion of the hip and comparing with the range of the healthy hip. Femoral anteversion may present with excessive internal rotation of the hip and lateral patella maltracking. Excessive femoral retroversion may be present with the opposite findings which can be examied clinically using Craig's test.

### 1.3 Radiological assessment

**Fig. 2:**
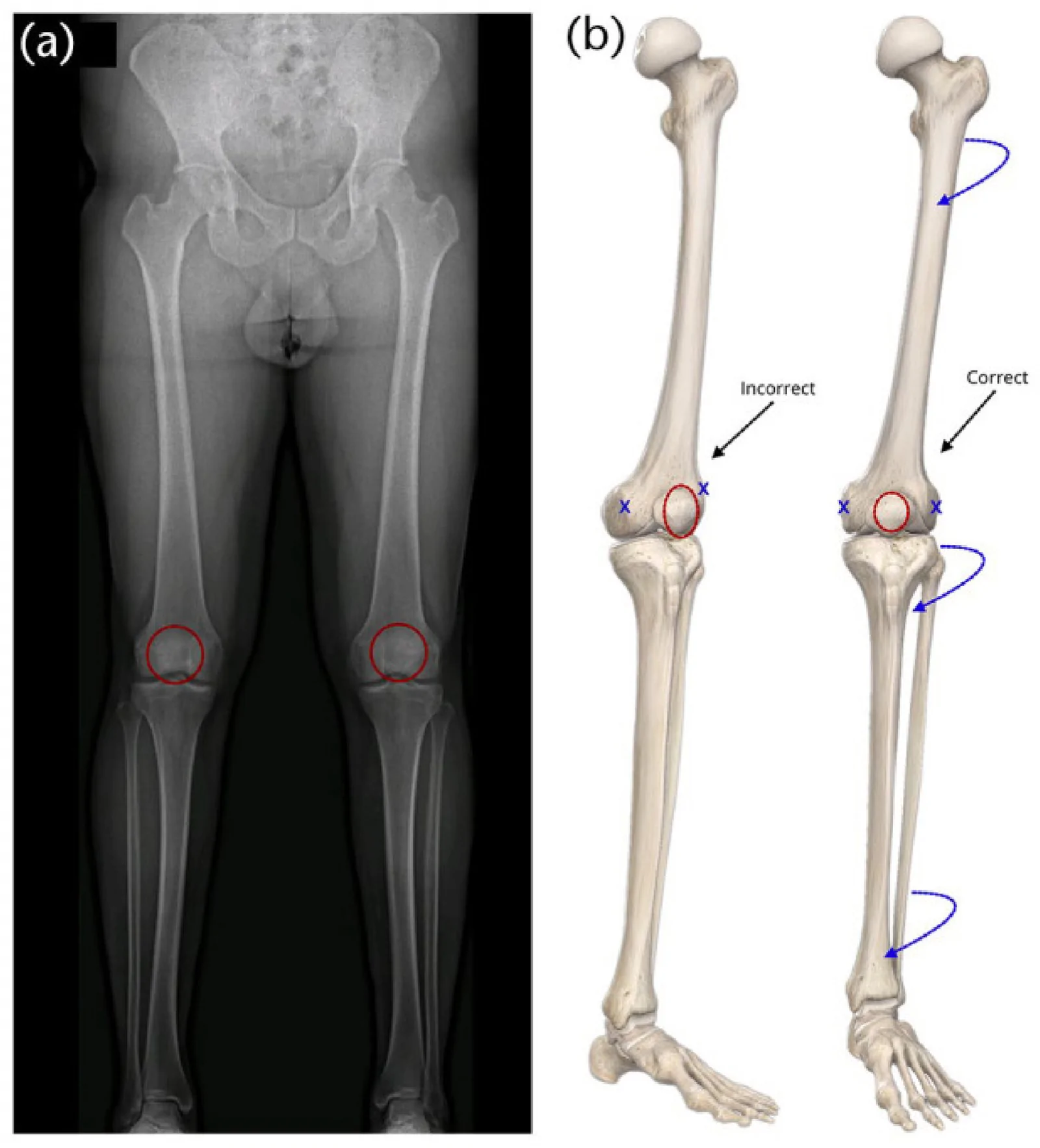
Long leg radiographs should be performed with patellae facing forward, knees in full extension, and the pelvis balanced with blocks under the short limb (or the long limb in cases with fixed equinus contracture).^23^.

Full length, weight bearing radiographs of both lower limbs are an important tool in understanding the overall limb alignment and mechanical axis.^19^ (Fig. 2). The mechanical axis is defined as the line from the femoral head to the most central and superior point of the intercondylar notch of the femur. The tibial mechanical axis is defined from the centre of the intercondylar notch to the defined point of the talus. The mechanical axis deviation (MAD) is defined as the distance between the center of the knee and the mechanical axis and can help assess coronal plane deformities. Assessing the hip version relative to the femoral condyles is vital. Long leg radiographs can also be supplemented with cross sectional CT imaging to better understand rotational profile.^20^

**Fig. 3:**
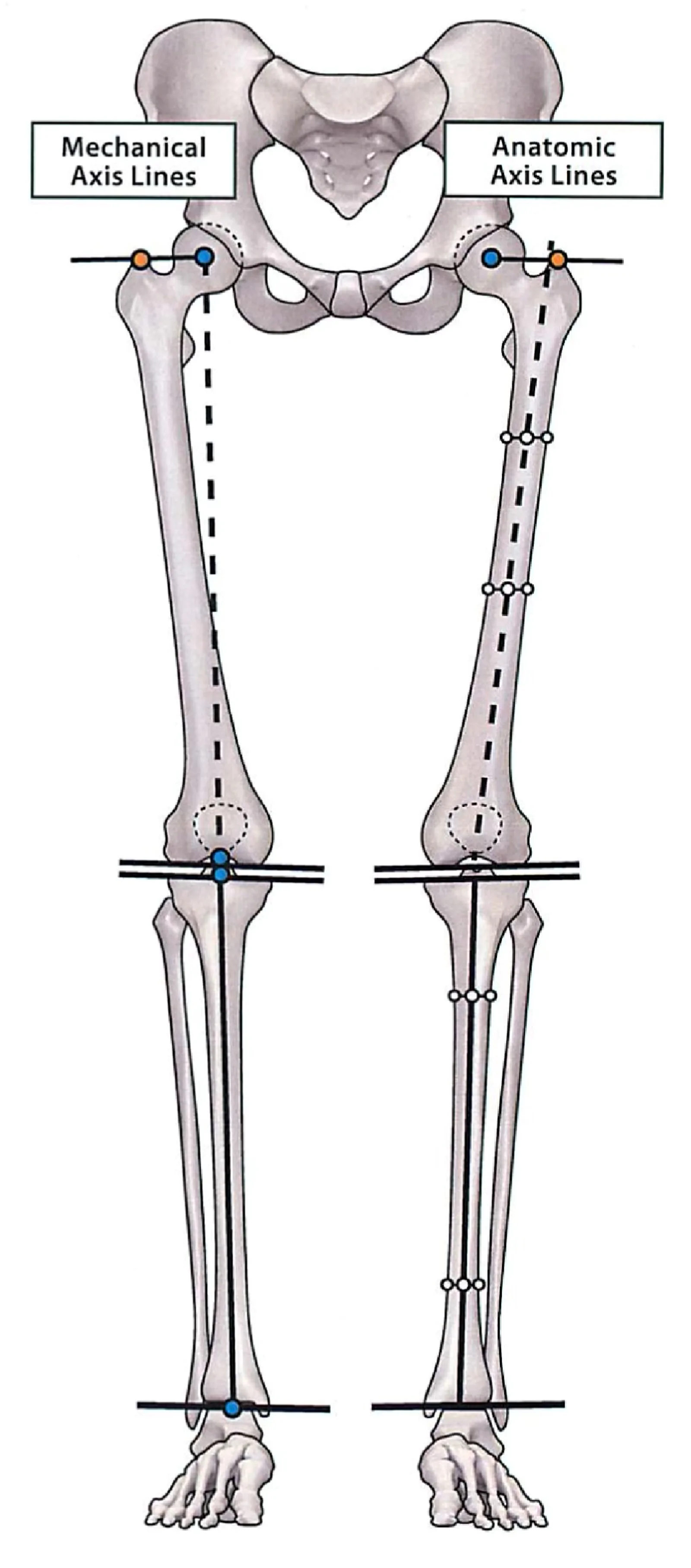
The femoral anatomic and mechanical axes differ. Tibial anatomic and mechanical axes are essentially the same.^23^.

**Fig. 4:**
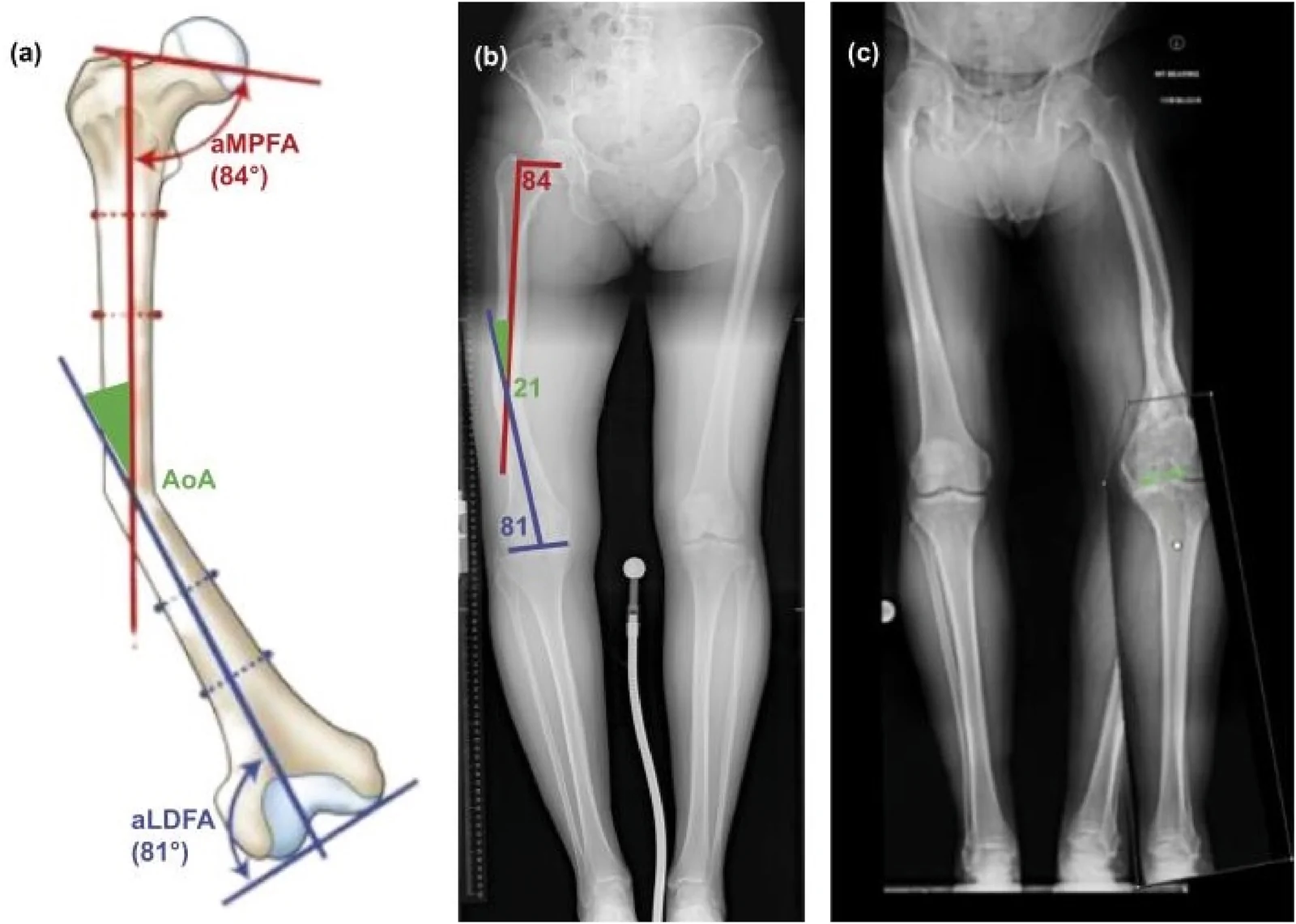
An example illustrating coronal assessment of deformity, based on population normal angles. (a) Depicts normal anatomic lateral distal femoral angle (aLDFA of 81°) and anatomic medial proximal femoral angle (aMPFA of 84°). The intersection of the proximal and distal anatomic axes is the apex of angulation (AoA). (b) Depicts a real-life “pen-and-paper" example of this method applied to a long leg radiograph..^24^ (c) Software deformity planning example.

However, when assessing the femoral deformity itself, the anatomic axis can be a simpler tool to use. The anatomic axis of the femur can be determined by using population normal angles at the level of the hip and knee, or the contralateral limb, if appropriate (see Fig. 3 and Fig. 4).^21^ ^22^

## 2 Treatment options

### 2.1 Non-operative treatment

Active non-operative treatment of femoral shaft malunions should aim to address functional needs and minimise the impact of the patient's deformity. Conservative management is best performed with a multidisciplinary approach, often involving orthotists, physiotherapy, occupational therapists, community nurses and the patient's support network. Non-operative may in fact require closer follow up to ensure that progress is being made.

#### 2.1.1 Consequences of non-operative management

If left untreated, femoral malunion can create an abnormal biomechanical environment. This can create secondary pathology proximally and distally to the malunion. Excessive anteversion may result in patello-femoral maltracking and/or femoro-actetabular impingement. This can also result in an internally rotated foot progression angle which can increase the risk of falls. Excessive retroversion can cause hip anterior labral impingement and early hip osteoarthritis.^25^ Leg length discrepancies may result in pelvic tilt and therefore abnormal femoral head coverage. Rotational malunion is the most poorly tolerated in terms of functional outcomes and the reported incidence after intramedullary nailing ranges from 19% to 56%.^26^ Tall et al. report other complications as well such as shortening, angulation, pain, secondary hip and knee osteoarthritis due to abnormal forces trough the joints.^27^^,^^28^ As such, operative mechanical axis corrections can be offered to address the functional deficits these patients experience.

### 2.2 Surgery

#### 2.2.1 Technical considerations

Corrective surgery for diaphyseal femoral malunion requires not only an accurate preoperative plan but also careful intraoperative execution, as even small technical errors can compromise the outcome. In the femur, osteotomy ideally at the apex of the deformity and acute correction is often feasible, but when it is not, gradual correction can be addressed with frame-assisted correction, Ilizarov with hinges or more commonly with hexapod frames.^6^^,^^29^ Several practical techniques help translate the planned correction into a reliable operative result. One of the most valuable resources available to the surgeon during malunion correction is the patient's own contralateral femur. Although preoperative CT provides objective measurement of torsion, the intraoperative environment does not always permit perfect reproduction of planned rotational angles. In these cases, visually referencing the contralateral (unaffected) limb becomes an intuitive and an effective tool. In practice, this involves comparing the projected profile of the lesser trochanter in relation to the femoral condyles under fluoroscopy, with matching patellar orientation. Taken together, these markers offer real-time feedback about rotation that complements the numerical targets established in the planning phase. This method is simple, reproducible, and highly dependable.

The quality of intraoperative imaging profoundly affects the precision of correction. Surgeons should strive for consistent, reproducible projections that truly represent the femoral neck and condylar axis. Poorly positioned AP or lateral views can mislead rotational assessment, especially following an osteotomy, when the distal segment is mobile. Many surgeons standardise their imaging checks using a stepwise routine:

- Confirm a true AP of the hip of the affected side
- Match the contralateral (unaffected) lesser trochanter profile
- Confirm contralateral (unaffected) knee AP appearance (patellar position)
- Match patella position on the affected side
- Obtain true lateral views of both knees
- Repeat final checks after locking the implant

#### 2.2.2 Nail or plate fixation

Intramedullary nailing remains a reliable method for managing diaphyseal femoral malunion, particularly when the deformity is primarily rotational or when stable axial alignment is required. Reamed nailing provides durable, load-sharing fixation and enhances the biological environment for union through endosteal stimulation. This has been shown to be particularly useful when utilising a transverse osteotomy and reamed intramedullary nailing in femoral malunions.^30^

Intramedullary nailing is especially useful when deformity is centred within the diaphysis and canal morphology permits controlled nail passage.^19^ The nail by design should fully correct angular diaphyseal deformities. Rotational correction can be performed accurately by aligning the proximal and distal segments before locking, and the intramedullary device maintains this torsional adjustment throughout healing.

Locking compression plates allow accurate angular correction and controlled compression across the osteotomy site. Plating is particularly beneficial when deformity is near a metaphyseal flare, where intramedullary passage is compromised, in the presence of implants for fracture fixation or joint replacement not allowing the insertion of an intramedullary nail, or when the shape the femur is irregular following correction. The latter occur when a polyapical deformity is corrected through a single osteotomy, or when the osteotomy is away from the apex of the deformity (osteotomy rule 2).^31^ Navadgi et al. reported reliable union and functional improvement using plate fixation for rotational and angular deformity.^32^

Diaphyseal thick cortical bone, does not behave the same way as metaphyseal bone intraoperatively. Performing a corrective osteotomy and maintaining an intact hinge is difficult and often unsuccessful. Therefore, when doing so, it is likely that the bone will be transected fully and become mobile. it is vital in this situation, not to introduce a new rotational or longitudinal deformity.

Hybrid constructs combining a plate with an intramedullary nail may offer improved stability in poor quality bone or where both axial and angular control are required.^33^

#### 2.2.3 External fixator

Circular ring fixators, including Ilizarov and traditional hexapod frames, remain powerful tools for gradual correction of multiplanar deformity, but they are poorly tolerated in the femur due to soft-tissue bulk, proximity to major muscle groups, psychological burden, and a high rate of pin-site irritations and stiffness.^28^^,^^32^ This reduced tolerance is well documented, with patients reporting significant functional and psychological impact even after device removal. Computerised hexapod systems allow highly accurate correction in all six degrees of freedom, yet their traditional use requires prolonged frame wear, which limits patient acceptance. Monolateral external fixators and rail systems are better tolerated than circular frames and offer simpler mechanics, but they may lack the fine multiplanar adjustability required for complex femoral deformities and share similar pin-site–related complications. Computerised hexapod systems allow highly accurate correction in all six degrees of freedom; importantly, they permit controlled, gradual correction, which may be advantageous in the presence of compromised soft tissues or extreme angular deformity where acute correction carries an unacceptable risk.^6^

#### 2.2.4 Fixator assisted correction

Once the osteotomy is performed, the limb becomes inherently unstable. Attempts to hold reduction manually during reaming or nail insertion often result in gradual drift or partial loss of correction. Temporary external fixation provides a stable, controllable platform that holds the corrected position while freeing the surgeon's hands for more precise technical work.

A simple unilateral frame can maintain alignment and prevent sagittal or rotational collapse. The 2 main advantages are that it preserves the correction while internal fixation is prepared, it allows incremental, fine adjustments before committing to definitive fixation. This technique is particularly useful in complex or multiplanar deformities where even slight deviation from the plan can lead to suboptimal results, the external fixator can be utilised as a reduction tool with appropriate pre-op planning.

A useful technique for controlling deformity during femoral osteotomy involves placing the external fixator half-pins “in chronic mode”—that is, intentionally inserting them *with the same angular deviation as the malunion itself*. By reproducing the deformity in the orientation of the pins, the surgeon can leverage the fixator as a real-time guide to achieve precise correction once the bone is osteotomized. This approach is particularly effective for rotational deformities. The technique can be carried out as follows:

- Before the osteotomy, the half-pins are inserted following the deformity, intentionally mirroring the magnitude and direction of the deformity.
- After the osteotomy, the pins are brought into true parallel alignment, thereby reducing the deformity in a controlled, mechanical manner.
- Once the corrected alignment is achieved, the limb can be held stable using the fixator while the intramedullary nail or plate is inserted.

**Fig. 5:**
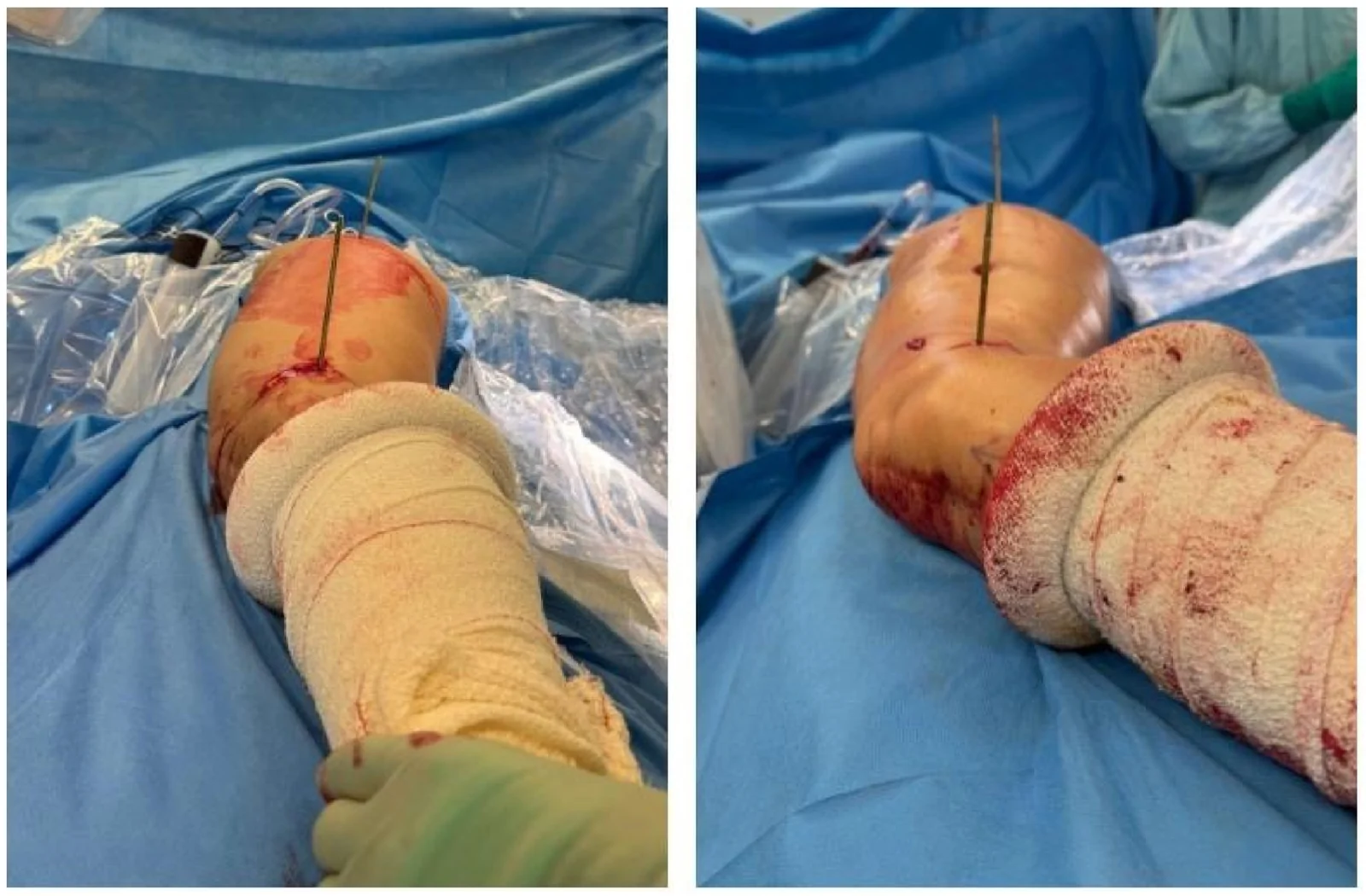
Half pins have been inserted into the femoral neck and along the intercondylar axis in a case of malunion with excessive anteversion.

By inserting the proximal and distal pins with the same divergent rotational angle as the malunion, the surgeon can subsequently de-rotate the limb until the pins become parallel, providing a simple and highly accurate intraoperative cue for restoring normal torsion (Fig. 5).

An osteotomy between these two points can then be performed, and the half pins can then be used to restore normal femoral version. When performing this method, it is important not to use thin bendable wires. Accurate changes can be made using electronic or manual goniometers intraoperatively.

**Fig. 6:**
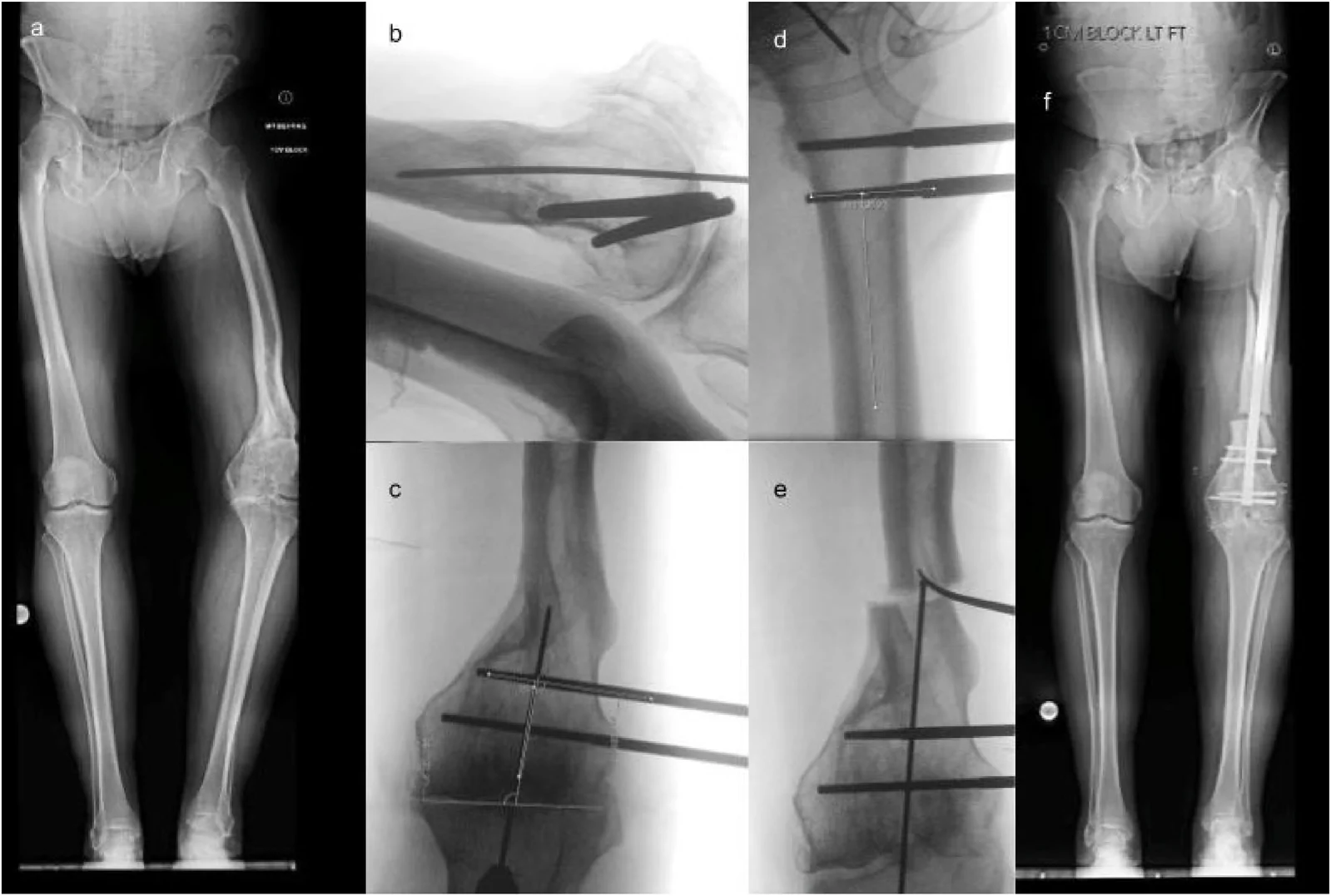
(A) preoperative deformity. (B and C) Distal half pin insertion, placed perpendicular to the anatomical axis and leaving space for the nail entry posteriorly. (D) Proximal half pins inserted perpendicular to the anatomical axis. (E) Osteotomy and correction. (F) Final deformity correction.

A similar strategy applies to coronal plane deformities (Fig. 6). The pins are placed such that their axes are coaxial with the angular deformity. Once the osteotomy is completed, the deformity is corrected by realigning these pins into a straight, colinear configuration. This converts a complex multiplanar deformity into a mechanically intuitive correction manoeuvre.

In both scenarios, the half-pins act not only as fixation points but also as visual and tactile alignment guides, ensuring that the correction achieved on the table accurately reflects the preoperative plan. This technique also stabilises the reduction during reaming or nail passage, reducing the risk of intraoperative drift and improving the precision of the final construct.

Modern techniques such as the CHAOS method—using a temporary intraoperative hexapod frame with definitive internal fixation—have evolved specifically to avoid the long-term morbidity associated with both ring fixators and monorail constructs while preserving the precision of multiplanar deformity correction.^34^^,^^35^

#### 2.2.5 Poller screws

Poller, or blocking, screws are an extremely effective adjunct in diaphyseal malunion surgery. In malunited femoral canals—often widened, eccentric, or irregular—the intramedullary nail may not track centrally, instead following the path of least resistance. This can unintentionally reintroduce deformity, particularly in the coronal plane.

Strategically placed poller screws act as “mechanical funnels,” guiding the nail down the intended path and preventing undesirable translation. They are especially valuable when:

- The original deformity involved varus or valgus angulation
- The medullary canal is asymmetric
- A reaming track from previous fixation biases the nail
- Bone stock is compromised

When combined with temporary external fixation, poller screws can entirely eliminate intraoperative drift during nail passage. Their placement requires minimal additional exposure but offers disproportionately large benefits in accuracy and stability.

#### 2.2.6 3D planning and patient-specific guides

Advances in 3D imaging and printing have significantly enhanced the precision of femoral malunion correction and a reduction in their cost. CT-based 3D reconstruction allows virtual simulation of osteotomy and correction. Patient-specific guides ensure accurate replication of planned osteotomy orientation and rotation. Oraa et al. reported correction within ±3° using PSI in diaphyseal malunions.^23^ D'Amelio et al. demonstrated reliable translational and angular accuracy with similar techniques.^36^^,^^37^ Oldhoff et al. validated these methods for femoral diaphyseal deformities, reporting improved accuracy compared with conventional techniques.^7^

While three-dimensional preoperative planning and patient-specific instrumentation can enhance the accuracy and reproducibility of deformity correction, their use may be constrained by increased cost, additional planning time, and the associated learning curve. Furthermore, access to advanced imaging, software platforms, and manufacturing of patient-specific guides may not be universally available, particularly in resource-limited settings. As such, while these technologies offer significant advantages in complex deformity correction, careful consideration of cost-effectiveness and institutional resources is required when selecting the most appropriate technique.

#### 2.2.7 Lengthening

To address true femoral shortening, limb lengthening can be achieved through distraction histogenesis, most commonly using a monolateral external fixator and, less frequently, a circular frame due to the bulk and limited tolerance of ring constructs around the femur.^19^^,^^38^

Where socioeconomic circumstances and healthcare resources permit, motorised intramedullary lengthening nails have increasingly been employed, allowing gradual limb lengthening without the need for an external device, albeit at substantially higher cost.

## 3 Summary

We have summarised our treatment options below in Table 1 to help aid decision making in these complex patients.

**Table 1:** A summary of the principal treatment strategies for femoral diaphyseal malunion and their typical advantages and limitations.

Treatment strategy	Indications	Advantages	Limitations
*Non-operative management*	Mild deformity. Minimal symptoms. High surgical risk.	Avoids operative morbidity (useful in frail patients).	Does not correct deformity. Risk of progressive biomechanical symptoms.
*Acute corrective osteotomy with internal fixation (nail or plate)*	Isolated angular or rotational deformity. Good bone stock. Deformity near diaphysis.	Immediate correction. Stable fixation. Early mobilisation	Limited capacity for large multiplanar deformities. Technically demanding. Risk of under/over correction.
*Gradual correction with external fixation (hexapod or Ilizarov frame)*	Severe multiplanar deformity. Large angular corrections. Soft tissue compromise.	Precise correction in multiple planes Gradual adjustment (can continue to adjust position to achieve optimal correction without further surgery)	Poor patient tolerance. Pin-site complications. Prolonged treatment.
*Fixator-assisted correction with internal fixation*	Complex deformities requiring precise intraoperative control but avoiding prolonged frame use.	Accurate correction Avoids long-term external fixation Improved intraoperative stability	Requires careful planning and technical expertise.
*3D planning with patient-specific guides*	Complex multiplanar deformity or rotational malalignment.	High correction accuracy Reproducibility	Increased cost. Limited availability.

## 4 Conclusion

Diaphyseal femoral malunion presents complex diagnostic and technical challenges. A systematic approach to clinical assessment, radiological evaluation, and deformity analysis remains fundamental to successful management. Although non-operative treatment may be appropriate in small magnitude deformities or minimally symptomatic patients, many cases warrant undergoing surgical correction to restore limb alignment and function.

Several key practical principles should guide management. Firstly, patient selection, a careful history and examination and multidisciplinary team working are all vital in preventing poor decisions being made. Crucially, long leg radiographs must be taken with the proper technique or can be quite misleading. The technique used to achieve the deformity correction should be individualised. Any number of techniques can be used to achieve the same goal. Successful management of femoral diaphyseal malunion depends less on the specific implant used and more on accurate deformity analysis, careful preoperative planning, and selecting the corrective technique that best matches the deformity pattern and patient factors.

## Patient consent

Informed Consent has been obtained from patient or guardian for the study's participation and publication of clinical pictures.

## Credit author

**Marco Pes:** Conceptualization; Methodology; Writing – Original Draft; Visualization.

**Yahya Ibrahim:** Methodology; Literature Review; Writing – Review & Editing; Validation, Corresponding Author.

**Nikhil Pandit:** Writing & Editing.

**Alexandros Vris:** Conceptualization; Project Administration; Writing – Review & Editing; Final Approval of Manuscript.

## Ethical statement

This article is a narrative review and did not involve human participants, identifiable personal data, or experimental procedures. Ethical approval was not required.

## Funding

None.

## Declaration of competing interest

The authors declare that they have no known competing financial interests or personal relationships that could have appeared to influence the work reported in this paper.
